# Effectiveness and Safety of STYLAGE^®^ L Lidocaine in the Treatment of Nasolabial Folds (NICE Study): A Randomized, Double-Blind, Split-Face Controlled Study

**DOI:** 10.1007/s00266-025-05105-2

**Published:** 2025-08-19

**Authors:** Sophie Converset-Viethel, Magdalena Dobosz, Martine Baspeyras, Kamila Skretkowicz-Szarmach, Kai-Uwe Schlaudraff

**Affiliations:** 1 Private Aesthetic Surgery Practice, Palais de Flore, 10 Boulevard Jules Favre, 69006 Lyon, France; 2 Private Dermatology Practice, Partyzantow 14/102, 80-254, Gdansk, Poland; 3 Private Dermatology Practice, 45, Avenue Bel Air, 33200 Bordeaux, France; 4 Private Dermatology Practice, Generala Augusta Emila Fieldorfa 21, 80-041 Gdansk, Poland; 5 Private Aesthetic Surgery Practice, Concept-Clinic, 24 Boulevard des Philosophes, Geneva, Switzerland

**Keywords:** Crossed-linked hyaluronic acid, Lidocaine, Dermal filler, Stylage, Nasolabial fold treatment, Randomized controlled trial (RCT)

## Abstract

**Background:**

Prominent nasolabial folds (NLFs) are a typical manifestation of aging. Hyaluronic acid (HA)-based injectable fillers are commonly used for correction. The NICE Study primarily evaluated non-inferiority of STYLAGE^®^ L Lidocaine (STYL-L), a cross-linked HA filler with lidocaine, versus the similar product Juvéderm^®^ ULTRA 3 (JUV-3) in correcting moderate to severe NLFs.

**Methods:**

This was a double-blind, randomized, controlled, within-subject (split-face) study with 12 months follow-up. The primary endpoint was mean change in Wrinkle Severity Rating Scale (WSRS) score from baseline to 6 months after treatment initiation. Secondary endpoints included differences in NLF depth using interference fringe projection profilometry, Global Aesthetic Improvement Scale (GAIS), subject satisfaction, pain during injection, and tolerance.

**Results:**

Of the 50 subjects randomized, 47 were analyzed. In the primary endpoint analysis, non-inferiority of STYL-L to JUV-3 was demonstrated as the upper limit of the 95% confidence interval (CI) was inferior to 0.5 (difference [95% CI] in WSRS change from baseline at 6 months was 0.11 [−0.07; 0.28]). NLF average depth was similarly reduced with STYL-L and JUV-3 at 1 month (adjusted mean [standard error] decrease of 0.049 [0.003] mm and 0.049 [0.004] mm, respectively), and generally maintained. Both evaluators and subjects considered NLFs improved, with all or almost all subjects classed as GAIS responders with both devices throughout the study. Overall subject satisfaction was high. Pain ratings were low. Both devices were well tolerated.

**Conclusions:**

Non-inferiority of STYL-L versus JUV-3 in improving NLFs was demonstrated. Results up to 12 months suggest comparable effectiveness and safety profiles.

**Level of Evidence I:**

This journal requires that authors assign a level of evidence to each article. For a full description of these Evidence-Based Medicine ratings, please refer to the Table of Contents or the online Instructions to Authors www.springer.com/00266.

## Introduction

Nasolabial folds (NLFs) are special anatomical entities. They form the boundary between the periorbital/lateral region of the cheek and perioral region, and act as a transition zone between the lateral malar and superficial perioral fat compartment. Anatomically, NLFs are fibrous zones crossed by the elevator muscles of the upper lip and the lateral commissures [1].

Prominent NLFs are a typical manifestation of facial aging. Subcutaneous fat and collagen loss leads to sagging of facial fat compartments and accentuated NLFs [2]. Injectable dermal fillers are a commonly used, minimally invasive, reversible method to correct NLFs with immediate results. Hyaluronic acid (HA)-based fillers are the most popular fillers [3]. HA is a naturally occurring, biodegradable glycosaminoglycan found in the skin with minimal immunogenic response [2, 4]. Long-term experience with HA fillers has shown them to be effective, durable, and well tolerated [3, 5]. There are several HA fillers with distinct characteristics (i.e., HA concentration, manufacturing technology, cross-linking specifications) [2, 6]. Cross-linking HA to agents such 1,4-butanediol diglycidyl ether (BDDE) protects against degradation and improves durability of the effect [6, 7]. A common complaint during treatment with fillers is injection pain; today many formulations include local anesthetics such as lidocaine hydrochloride to improve patient comfort [8, 9].

STYLAGE^®^ L Lidocaine is a cross-linked HA filler with lidocaine designed to replenish facial volume and correct skin depressions. The present double-blind, randomized, within-subject, non-inferiority study (NICE Study) compared STYLAGE^®^ L Lidocaine to Juvéderm^®^ ULTRA 3. Both devices have European Conformity (EC) marking since 2012 and 2007, respectively. The comparator was chosen for its similar formulation and composition (cross-linked HA with 0.3% lidocaine), significant clinical record of safety and effectiveness, and similar intended use. This study primarily evaluated the effectiveness of two different fillers in correcting moderate to severe NLFs using the validated Wrinkle Severity Rating Scale (WSRS) [10]. The WSRS has been widely used in studies evaluating effectiveness of HA fillers in correcting NLFs [4, 11–14]. In addition, NLF depth was objectively measured using a fringe projection system (DermaTOP^®^) which reconstitutes a 3D image of the skin surface [15].

## Materials and Methods

### Study Design

This was a prospective, double-blind, randomized, controlled, within-subject, multicenter study conducted in 2 centers (Eurofins-Dermscan, 1 center in Poland and 1 in France) from 16 April 2021 to 06 July 2022. It was conducted in compliance with Good Clinical Practice, fulfilling International Organization for Standardization 14155:2020, and in accordance with the Declaration of Helsinki and local laws. Informed consent was obtained from each subject before study admission. The study received ethics committee approval prior to commencement. Subjects were not involved in study design/conduct/reporting.

### Population

Healthy male or female subjects, aged 30 to 65 years, with symmetrical moderate to severe NLFs (assessed live as Grade 3 or 4 on both sides using the WSRS) were eligible for inclusion. The main inclusion/exclusion criteria are provided in Table 1. Subjects provided informed consent, including agreeing to facial photographs.

**Table 1:** Main inclusion and exclusion criteria

*Main inclusion criteria*
• Healthy female or male between 30 and 65 years. • With approximate symmetry, moderate to severe NLFs attaining either Grade 3 on both sides or Grade 4 on both sides on the WSRS for NLF, as assessed in live. • With marionette’s lines that do not require to be treated according to investigator. • Agreeing not to receive another aesthetic procedure on the face (e.g., laser, dermabrasion, surgery, deep chemical peeling, surface peel, tensor threads, injection with a filling product) during the whole study.
*Main exclusion criteria*
General conditions: • With tendency to develop hypertrophic or keloid scars. • With known hypersensitivity to study products or history of severe or evolutive / unstable / recent allergy. • With inflammation or infectious cutaneous disorders in or near the studied zones (herpes, acne, mycosis, papilloma, …). • With history of streptococcal disease, such as acute rheumatic fever or recurrent sore throats and in case of acute rheumatic fever with heart complications. • With history or ongoing autoimmune disease and/or immune deficiency. • With known porphyria or cardiac conduction disorders, or with untreated epilepsy. Previous treatments: • Under oral surgery (e.g., tooth extraction, orthodontia, or implantation) within 6 weeks prior to screening visit or who plans to undergo any of these procedures during the study. • Under treatment with a surface peel below the inferior orbital rim within the past 6 months prior to screening visit. • Under treatment with a laser, a dermabrasion, a surgery, a deep chemical peeling, or other ablative procedure, or with a resorbable filling product (e.g., hyaluronic acid, collagen) below the inferior orbital rim within 12 months prior to screening visit. • Under treatment at any time with tensor threads below the inferior orbital rim, or with a slowly resorbable or non-resorbable permanent filling product.

*NLF* Nasolabial fold; *WSRS* Wrinkle Severity Rating Scale.

### Intervention and Comparator

STYLAGE^®^ L Lidocaine (STYL-L; Laboratoires VIVACY, Archamps, France) was the investigational device. Juvéderm^®^ ULTRA 3 (JUV-3; ALLERGAN, Annecy, France) was the active comparator. Both are injectable, resorbable, cross-linked HA gels (24 mg/g) with lidocaine hydrochloride (3 mg/g). STYL-L is cross-linked with BDDE using Inter-Penetrated Networks-Like^®^ (IPN-Like^®^) cross-linking technology while JUV-3 is cross-linked with BDDE using Hylacross technology [16].

The study used a ‘split-face’ design where subjects received both devices in opposite NLFs. Subjects received treatment at baseline. A touch-up session was performed 1 month later if deemed necessary by the subject and injector. Linear retrotracing or multipoint injection technique, or a combination of both, was used at the injector’s discretion. A 27½G needle or 22G/25G cannula was used. Volume was determined by the injector (maximum total of 2 mL/NLF for first injection, and 1 mL/NLF at optional touch-up).

### Randomization and Blinding

Live evaluators enrolled and assigned preselection/randomization numbers according to chronological order of arrival in study/randomization. Subjects received STYL-L at baseline in either the right or left NLF and JUV-3 in the opposite NLF. The side of the face and order of device injection (first or second) were according to a randomization list generated by Inferential (France) before study start.

The injectors had significant previous experience with both devices. Subjects were blindfolded during injection. Evaluators were independent. Live evaluators were different from the photographic evaluator, and all were blinded to the filler device used. The photographic evaluator was also blinded to visit.

### Study Outcomes and Measures

The primary objective was to demonstrate the non-inferiority of STYL-L versus JUV-3 in the correction of NLFs. Effectiveness was assessed 1, 3, 6, 9, and 12 months after the first injection session. The primary endpoint was mean change in NLF severity from baseline to 6 months after first injection assessed by live evaluators using the 5-grade WSRS (Grade 1 = absent; Grade 2 = mild; Grade 3 = moderate; Grade 4 = severe; Grade 5 = extreme) [10].

Mean WSRS changes from baseline to all time points after the first injection were assessed by live evaluators and using photographs. The ColorFace^©^ acquisition system (Newtone Technologies) was used for standardized photographs. WSRS responder rates (subjects with at least 1-point improvement from baseline for live evaluations), were assessed at all time points. Other secondary endpoints included Global Aesthetic Improvement Scale (GAIS) score and responder rates assessed by subjects and live evaluators at all time points. The GAIS is a validated 5-point rating scale evaluating post-treatment aesthetic improvement (1 = very much improved; to 5 = worse). Responders were subjects with a rating of ‘improved’, ‘much improved’ or ‘very much improved’. NLF depth, using DermaTOP^®^ (Breuckmann), was collected at the French center at all time points. Subject satisfaction using a questionnaire (4 questions) was collected throughout the study.

Tolerance was assessed throughout the study by collection of adverse events (AEs), injection site reactions (ISRs) (by live evaluators at each time point and by subject’s daily diary until 1 month after last injection and thereafter, if present, spontaneously). Subjects rated pain during injection on an 11-point scale (0 = no pain; to 10 = worst possible pain).

### Sample Size Calculation and Statistical Analysis

A minimum of 39 subjects were required for a 1-sided paired test at a 2.5% significance level to detect a 0.5 non-inferiority with a power of 90%, assuming the difference between groups in WSRS change from baseline at 6 months was equal to 0, and a standard deviation (SD) of 0.9. Assuming a dropout rate of approximately 20% at 6 months, 50 subjects were planned to be included in the study.

#### Primary Endpoint Analysis

A Mixed Model for Repeated Measurements (MMRM) was used, including device and time point as fixed factors, device by time point interaction and WSRS score at baseline as a covariate, and subject as a random factor. Device comparisons were done by means of the contrasts on the treatment factor by time effect. STYL-L was considered non-inferior to JUV-3 if the upper limit of the two-sided 95% confidence interval (CI) of the difference (STYL-L minus JUV-3) was inferior or equal to 0.5. If non-inferiority was demonstrated, superiority of STYL-L versus JUV-3 was evaluated. Superiority was achieved if the upper limit of the two-sided 95% CI of the difference was inferior to 0.

A supportive analysis using multiple imputation for missing values was planned but there were no missing data in the per-protocol (PP) population.

#### Secondary Endpoint Analyses

WSRS and GAIS scores, and NLF depth were compared between devices using the same methods as the primary endpoint analysis. Mean pain scores were compared between devices using Student’s t-test or Wilcoxon signed-rank test for paired samples. Descriptive statistics were presented for subject satisfaction. AEs were coded using MedDRA v24.0. Safety data were analyzed descriptively.

Inferential (France) performed data management, sample size calculation, and statistical analyses.

## Results

### Recruitment and Subject Characteristics

**Fig. 1:**
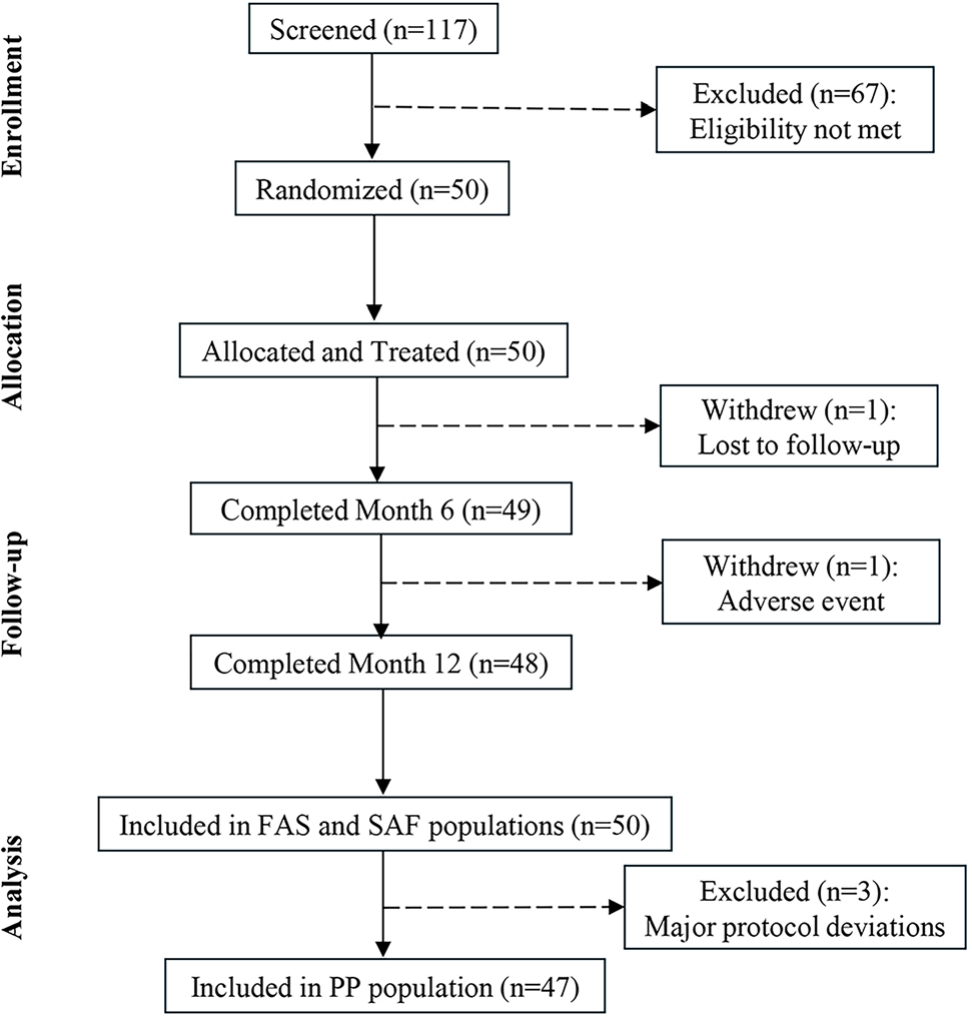
Consort flow diagram and analysis populations. *FAS* Full analysis set; *n* Number of subjects; *PP* Per-protocol; *SAF* Safety (population). The SAF population included all subjects who received at least one treatment with a study device. This population was used for analysis of baseline and safety data. The FAS population included all randomized subjects who received at least one treatment with a study device. This population was used for the analysis of the primary and secondary effectiveness endpoints. The PP population included all subjects from the FAS who met all inclusion/exclusion criteria and who completed the 6-month follow-up visit (i.e., subjects who answered the primary effectiveness endpoint) with no major protocol deviations that could impact the primary endpoint. This was the primary population for effectiveness analysis

Of the 117 subjects screened, 50 were eligible for study inclusion, randomized, and treated (25 subjects in each of the 2 centers) (Fig. 1). All injections were administered according to the randomization list, except for 1 subject who received devices in opposite NLFs to those planned. A total of 49 subjects completed the 6-month follow-up and 48 the 12-month. The PP was the primary population for effectiveness analyses (N=47). Most randomized treated subjects were female (48 [96.0%] subjects) with Grade 3 (Moderate) NLFs (70.0%) at baseline (Table 2). Mean age (range) was 50.8 (32–65) years.

**Table 2:** Subject baseline characteristics - Safety population (N=50)

	All subjects (N=50)
*Sex, n (%)*	
Male	2 (4.0)
Female	48 (96.0)
*Age (years)*	
Mean (SD)	50.8 (9.08)
Median (Min; Max)	51.5 (32; 65)
*WSRS assessed by live evaluators*^*a*^	
*Severity, n (%)*	
Grade 3 (Moderate)	35 (70.0)
Grade 4 (Severe)	15 (30.0)
*Score*	
Mean (SD)	3.30 (0.463)
Median (Min; Max)	3.00 (3.0; 4.0)

*Max* Maximum; *Min* Minimum; *N* Number of subjects in the population; *n* number of subjects; *SD* Standard deviation; *WSRS* Wrinkle Severity Rating Scale.

^a^ Identical grade for both device groups.

### Injection Characteristics

At baseline, all subjects received injections with both devices. The mean (SD) volume injected was 1.05 (0.231) mL with STYL-L and 1.01 (0.225) mL with JUV-3. Approximately half of the subjects received the optional touch-up (22 [45.8%] with STYL-L and 23 [47.9%] with JUV-3, mean 0.47 mL with both devices). Most injectors used the linear retrotracing technique with cannula at both injection sessions.

### Effectiveness

#### WSRS

**Fig. 2:**
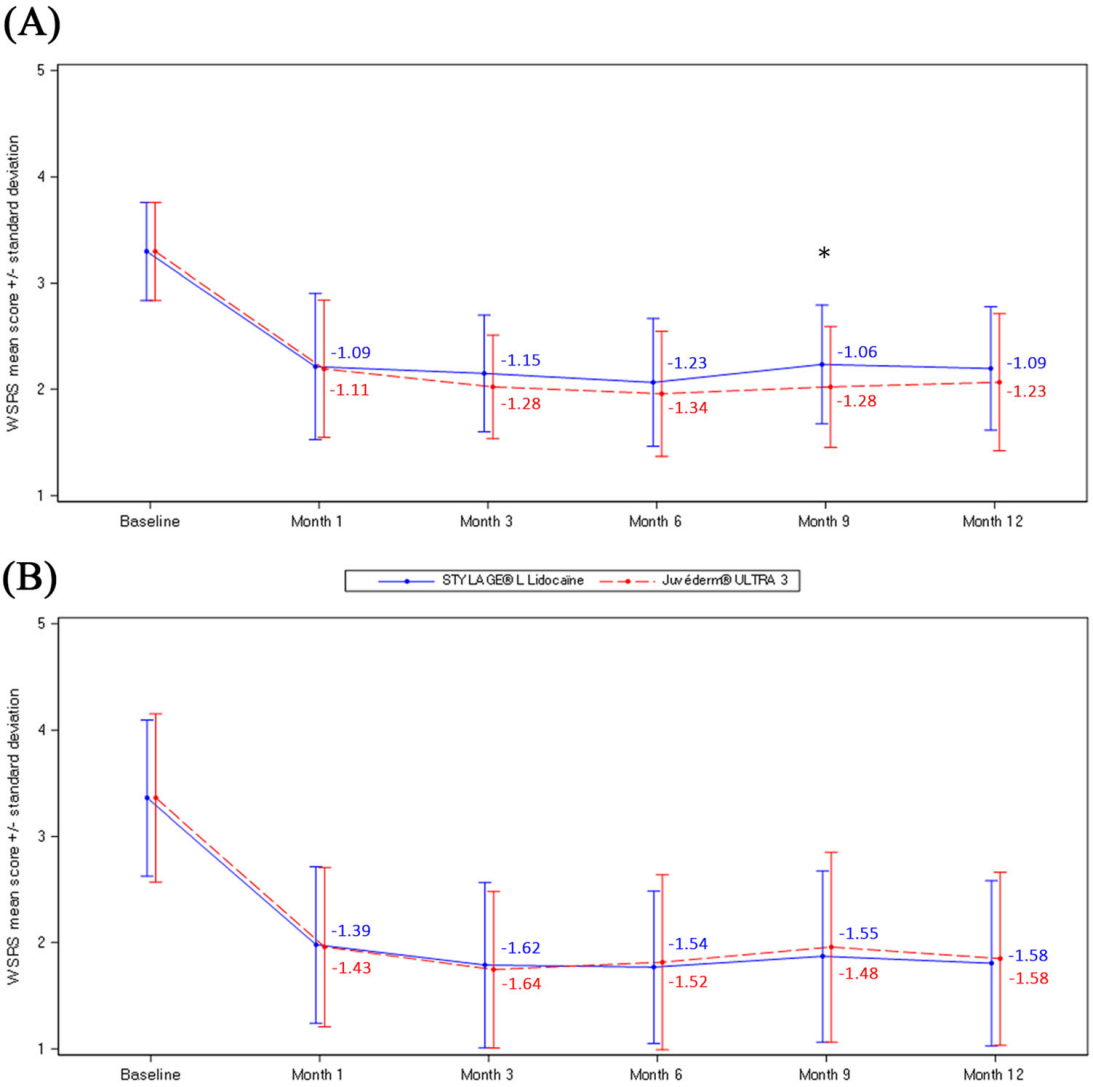
Mean WSRS score by visit assessed by **A** live independent blinded evaluators and **B** an independent blinded evaluator using photographs – PP population (N=47). *N* Number of subjects in the population; *PP* Per-protocol; *WSRS*, Wrinkle Severity Rating Scale. Values in figure are the adjusted mean change from baseline, * *p*<0.05 for difference between devices in mean change from baseline

In the MMRM analysis of the primary endpoint, the adjusted mean (standard error, SE) change from baseline at 6 months was -1.23 (0.085) and -1.34 (0.084) with STYL-L and JUV-3, respectively (Fig. 2A). The difference [95% CI] was 0.11 [-0.07; 0.28]. As the upper limit of the 95% CI was inferior to 0.5, the non-inferiority of STYL-L to JUV-3 was demonstrated. Superiority was not demonstrated as the upper limit was superior to 0.

When considering all live evaluation time points (Fig. 2A), mean WSRS scores decreased notably and similarly with both devices 1 month after first injection and decreased further at 3 and 6 months. Scores increased slightly at 9 and 12 months compared to 6 months, with the difference [95% CI] between devices being statistically significant in favor of JUV-3 at 9 months (0.21 [0.03; 0.39], *p*=0.020) (differences 0.02 to 0.14, *p* values 0.074 to 0.829 at other time points). In photographic evaluations (Fig. 2B), mean WSRS scores decreased notably and similarly with both devices 1 month after first injection and decreased further at 3 months. Scores increased slightly at 6 months and were maintained until 12 months. There was no statistically significant difference between devices at any time point (differences -0.07 to 0.04, *p* values 0.468 to 0.999).

**Fig. 3:**
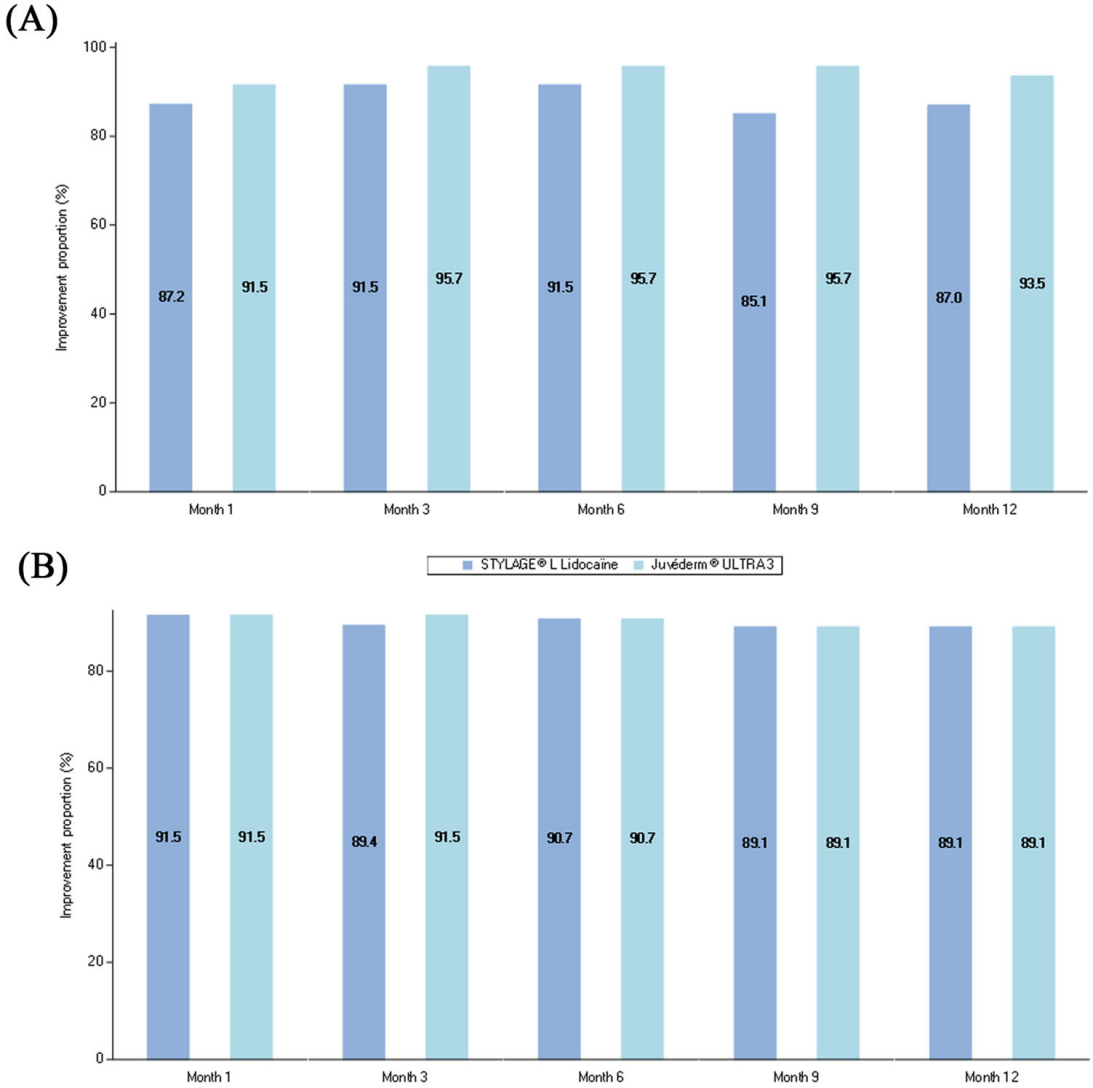
Proportion of improved subjects (responders) on the WSRS by visit assessed by **A** live independent blinded evaluators and **B** an independent blinded evaluator using photographs - PP population (N=47). *N* Number of subjects in the population; *PP* Per-protocol; *WSRS* Wrinkle Severity Rating Scale

According to the live evaluation, the vast majority of subjects were responders with both devices at 1 month (41 [87.2%] and 43 [91.5%] subjects with STYL-L and JUV-3, respectively) (Fig. 3A). The proportion of responders remained similar up until 12 months with no statistical difference between devices at any time point (differences −10.6 to −4.3, *p* values 0.059 to 0.527). Similar results were observed in the photographic evaluations (differences -2.1 to 0, *p* values 0.705 to 1) (Figure 3B).

**Fig. 4:**
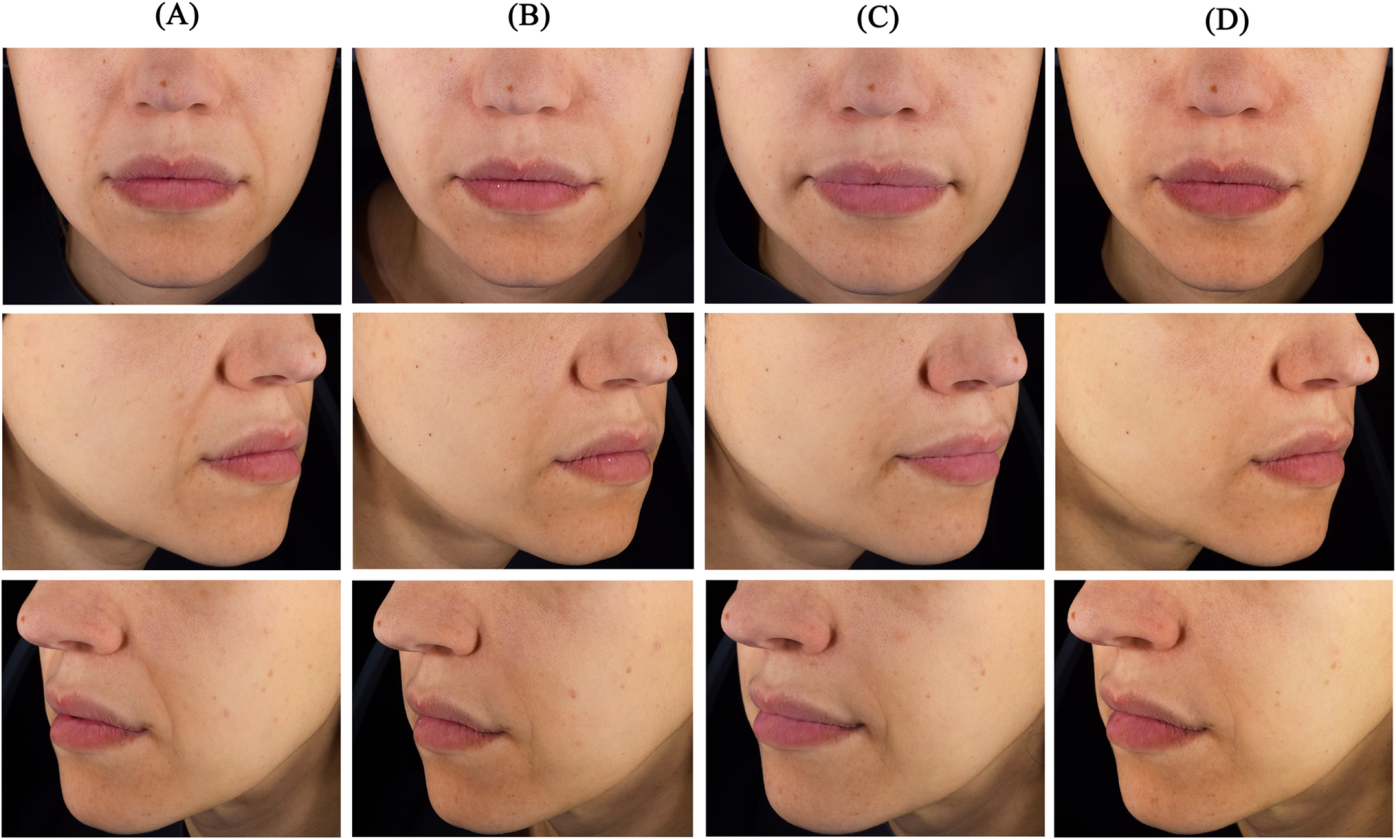
NLF appearance of a 33-year-old woman at **A** baseline, and at **B** 3 months, **C** 6 months, and **D** 12 months after injection with Juvéderm^®^ ULTRA 3 in the right NLF and STYLAGE^®^ L Lidocaine in the left NLF. NLF, Nasolabial fold. 1 mL of Juvéderm^®^ ULTRA 3 and 1 mL of STYLAGE^®^ L Lidocaine were injected in the right and left NLF, respectively, using a linear retrotracing technique with a 25G cannula. No touch-up injection was performed at 1 month. Upper, middle, and lower photographs correspond to front, right and left 45° profiles, respectively

**Fig. 5:**
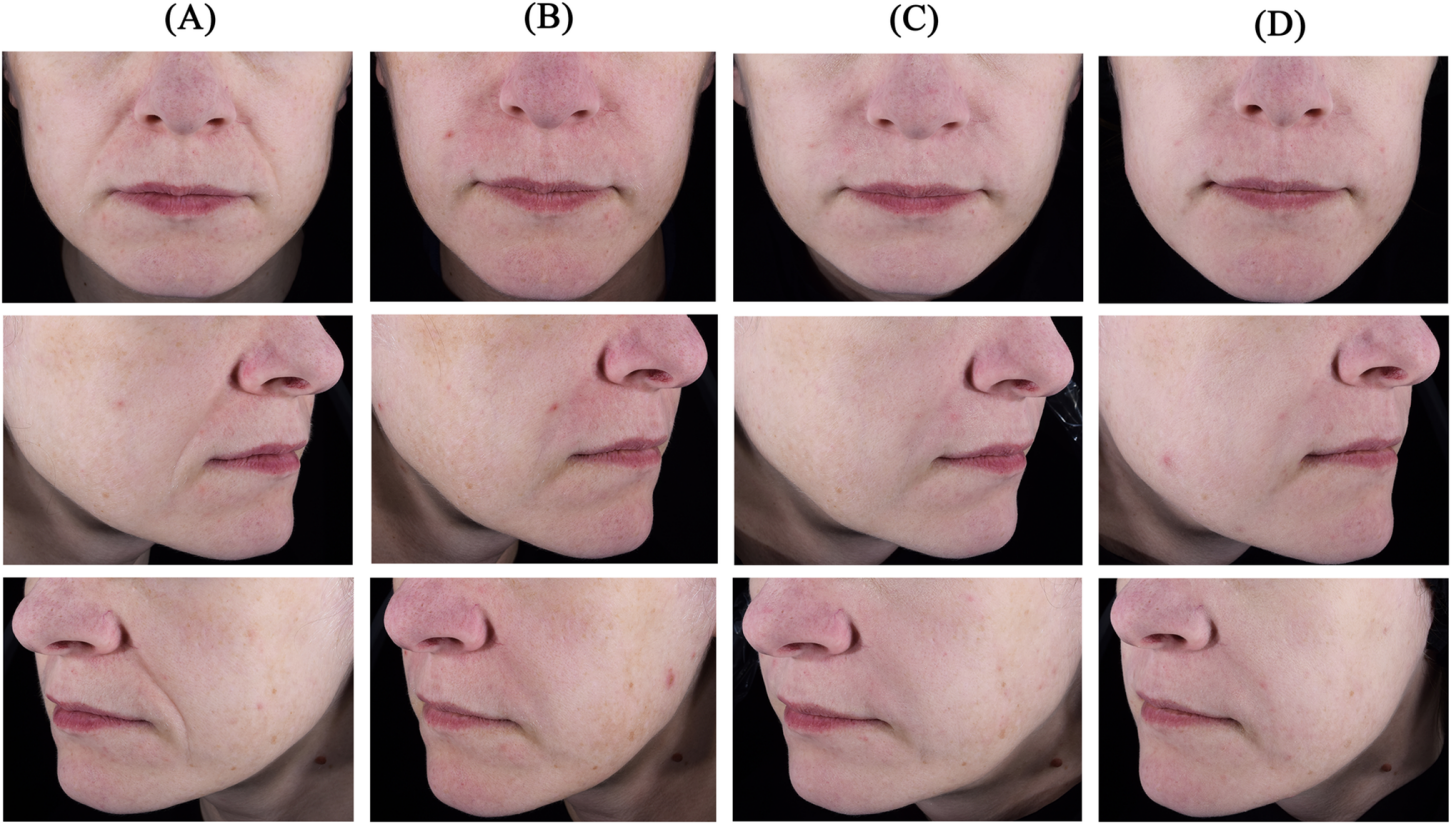
NLF appearance of a 39-year-old woman at **A** baseline, and at **B** 3 months, **C** 6 months, and **D** 12 months after injection with Juvéderm^®^ ULTRA 3 in the right NLF and STYLAGE^®^ L Lidocaine in the left NLF. *NLF*, Nasolabial fold. 1 mL of Juvéderm^®^ ULTRA 3 and 1 mL of STYLAGE^®^ L Lidocaine were initially injected at baseline in the right and left NLF, respectively. A touch-up with an injection of 0.2 mL of STYLAGE^®^ L Lidocaine was done in the left NLF 1 month after. All injections were done using a multipoint technique with a 27½G needle. Upper, middle, and lower photographs correspond to front, right and left 45° profiles, respectively

Figures 4 and 5 illustrate the improvement in NLF severity.

#### NLF Average Depth - DermaTOP^®^

**Fig. 6:**
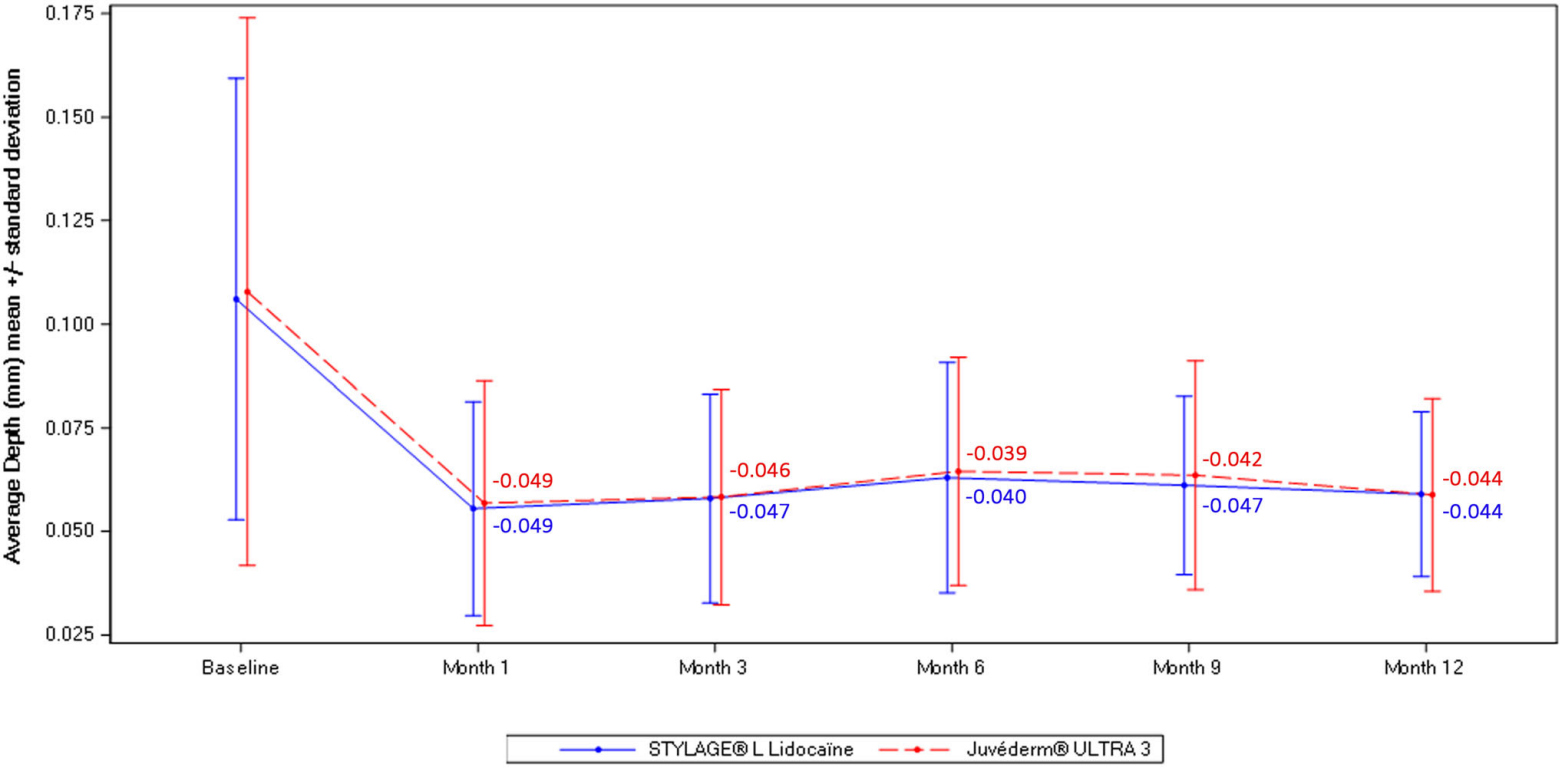
Quantification of NLF average depth by visit using DermaTOP^®^ – Subjects from French center in PP population (N=23). *N*, Number of subjects in the population; *NLF*, Nasolabial fold; *PP* Per-protocol. Values in figure are the adjusted mean change from baseline

NLF average depth (Fig. 6) was notably and similarly reduced after first injection with both devices at 1 month (adjusted mean [SE] decrease of 0.049 [0.003] mm and 0.049 [0.004] mm with STYL-L and JUV-3, respectively), and generally maintained up until 12 months (adjusted mean [SE] decrease of 0.044 [0.003] mm with both devices). Differences between devices were not statistically significant.

#### Subject Satisfaction

Global satisfaction (‘somewhat satisfied’, ‘satisfied’ and ‘very satisfied’) was high for all questions with both devices at all time points. Over 95% of subjects were globally satisfied with how their NLFs looked without expression and when they smiled, and how natural the NLFs looked. Over 93% found their NLFs aesthetically pleasing.

#### Pain During Injection

Mean (SD) ratings of pain during injection were low and similar with both devices at first injection (2.5 [1.8] and 2.6 [1.7] out of 10 with STYL-L and JUV-3, respectively, *p*=0.989), and the optional touch-up (2.6 [1.4] and 2.3 [1.3], respectively, *p*=0.727).

#### GAIS

**Fig. 7:**
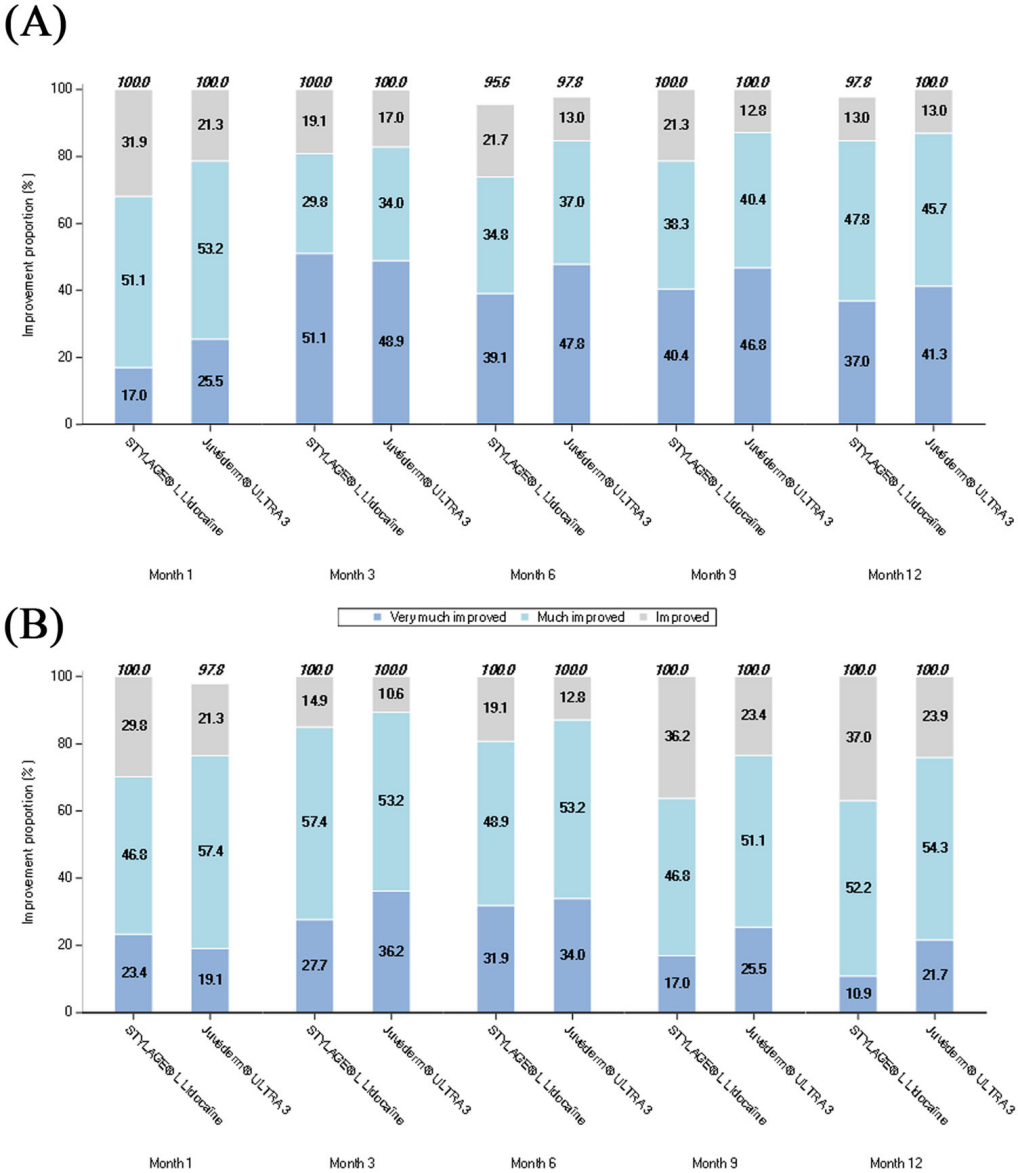
Proportion of improved subjects (responders) on the GAIS by visit assessed by **A** the subjects and **B** live independent blinded evaluators – PP population (N=47). *GAIS* Global Aesthetic Improvement Scale; *N* Number of subjects in the population; *PP* Per-protocol

All 47 subjects considered themselves responders to both devices at 1 and 3 months (Fig. 7A). The proportion remained high and similar up until 12 months. The live evaluator assessment was broadly in alignment (Fig. 7B). Differences between devices were not statistically significant, or non-evaluable where all subjects were improved. GAIS mean score was generally comparable between devices over the study. Statistical significance in favor of JUV-3 was reached for isolated time points (difference [95% CI] at 1 and 6 months was −0.2 [−0.4; 0.0], *p*=0.044, and −0.2 [−0.4; −0.1], *p*=0.008, respectively) in the subject assessment.

### Safety Analysis

Both devices were generally well tolerated.

Evaluator-reported ISRs were mostly redness/erythema and swelling/edema, and predominantly mild in intensity. The only ISRs ongoing at 3 months and beyond were mild and were swelling/edema (in 1 subject with STYL-L at 3 months), induration (in 2 subjects with STYL-L and 1 with JUV-3 at 3 months), and redness/erythema (in 1 subject with each device at 3, 6 and 12 months, and in 1 subject with STYL-L at 9 months). The proportion of subject-reported ISRs was similar between devices (90.0% and 86.0% with STYL-L and JUV-3, respectively) and were mostly mild or moderate in intensity. ISRs were mostly pain/tenderness, lumps/bumps, and swelling/edema with both devices. When considering individual ISRs, most lasted ≤7 days except for lumps/bumps and induration.

The incidence of device-related AEs (adverse device effects) was slightly higher with STYL-L than with JUV-3 but still generally low (16 [32.0%] subjects with 19 events versus 10 [20.0%] subjects with 11 events) (Table 3). Device-related AEs were predominantly following ISRs, with induration being the most common with both devices. Most events were mild in intensity, with 1 moderate and 1 severe event related to STYL-L.

**Table 3:** Device-related AEs by system organ class and preferred term - Safety population (N=50)

	STYLAGE^®^ L Lidocaine (N=50)		Juvéderm^®^ ULTRA 3 (N=50)		Other (N=50)	
	n (%)	e	n (%)	e	n (%)	e
At least one device-related AE	16 (32.0%)	19	10 (20.0%)	11	2 (4.0%)	2
**General disorders and administration site conditions**	**16 (32.0%)**	**18**	**10 (20.0%)**	**11**	**1 (2.0%)**	**1**
Injection site induration	11 (22.0%)	11	7 (14.0%)	7	0 (0.0%)	0
Injection site mass	2 (4.0%)	2	1 (2.0%)	1	0 (0.0%)	0
Injection site pain	2 (4.0%)	2	1 (2.0%)	1	0 (0.0%)	0
Injection site telangiectasia	1 (2.0%)	1	0 (0.0%)	0	1 (2.0%)	1
Injection site discolouration	1 (2.0%)	1	0 (0.0%)	0	0 (0.0%)	0
Injection site erythema	0 (0.0%)	0	1 (2.0%)	2	0 (0.0%)	0
njection site edema	1 (2.0%)	1	0 (0.0%)	0	0 (0.0%)	0
**Gastrointestinal disorders**	**0 (0.0%)**	**0**	**0 (0.0%)**	**0**	**1 (2.0%)**	**1**
Aphthous ulcer	0 (0.0%)	0	0 (0.0%)	0	1 (2.0%)	1
**Skin and subcutaneous tissue disorders**	**1 (2.0%)**	**1**	**0 (0.0%)**	**0**	**0 (0.0%)**	**0**
Skin wrinkling	1 (2.0%)	1	0 (0.0%)	0	0 (0.0%)	0

*AE* Adverse event; *e* Number of device-related AEs; *ISR* Injection site reaction; *N* Number of subjects in the population; *n* Number of subjects with at least one device-related AE; *%* (n row / N column) × 100. Data related to System Organ Class are highlighted in bold characters.

An ISR was reported as a device-related AE if a concomitant treatment was required, if the event needed follow-up by the evaluator, or if the event was still present at the touch-up visit or at least 1 month after the touch-up injection, if applicable, regardless of the actual ISR start date. Device-related AEs were those with at least one episode categorized by the evaluator as 'probably related', 'possibly related', or 'causal relationship' with the device or with the relationship to the device missing. AEs considered as related to the device but not to one treatment specifically (i.e., AEs related to both treatments) are accounted for in the ‘Other’ column. The event of aphthous ulcer considered possibly related to the device (device was not specified) was also considered possibly related to study procedure due to stress before injection.

## Discussion

The NICE study demonstrates that STYL-L and JUV-3 have comparable effectiveness. With both devices WSRS scores notably improved at 1 month and were generally sustained up to 12 months. There was a slight reversal around 6 months likely due to the resorbable nature of HA gels. However, WSRS scores remained well below baseline at all time points. Statistical significance in favor of JUV-3 was reached for an isolated time point (at 9 months) in the live evaluation but this was not confirmed by the photographic evaluation. Clinical observations have shown that beneficial effect is maintained beyond the expected longevity of the HA filler [17]. There is evidence that the mechanical pressure exerted by the filler within the dermis stimulates collagen production and up-regulates elastin [17, 18], which may explain the long-term effect observed with both devices in this study.

In the current study, mean WSRS improvements of 1.23 and 1.34 were observed with STYL-L and JUV-3, respectively, at 6 months. Similar improvements were observed in a shorter duration split-face study using JUV-3 (known as Juvéderm^®^ ULTRA PLUS in the US) as a comparator [19]. A cross-linked HA filler was considered non-inferior to JUV-3: mean (SD) WSRS score improved by 1.1 (0.75) and 1.1 (0.68), respectively, at 24 weeks (primary endpoint) and generally sustained up to 48 weeks.

The WSRS is a validated, widely used tool, specifically designed to assess NLF severity [10]. The notion of stretching the NLF during assessment is mentioned in the standardized grade definitions. However, in the literature, the WSRS has been used for live and photographic evaluations in combination [20–22] as in the current study. In Choi et al. (2020) [12] and Lee et al. (2017) [13], as well as the Day et al. (2004) WSRS validation study [10], only photographs were used. Both front and 45° profile photographs were taken in the NICE study for evaluation by blinded evaluators. Taking profile photographs into account may give a more accurate representation of the length and, more specifically, depth of NLFs. Comparing WSRS scoring by the same evaluators with and without profile photographs could be of particular interest and may lead to a refined analytical tool. The use of standardized imaging, such as the ColorFace^©^ system used in the current study, is important to ensure reproducible positioning of the subject in such situations.

The WSRS results of the NICE study were supported by quantitative interference fringe projection profilometry analysis using DermaTOP^®^, which showed improvement in NLF average depth with both devices in line with the subjective WSRS assessment. The interest in this objective measure of NLF correction in clinical studies is increasing, with various high-resolution systems available such as DermaTOP^®^, Primos^®^ and Skinstation^®^ [14, 21, 23, 24]. These systems provide greater accuracy in assessing NLF depth and volume and serve as a useful supportive measure to the widely used WSRS. The GAIS results demonstrated that both evaluators and subjects considered NLFs improved, with all or almost all subjects classed as responders with both devices throughout the study. Overall subject satisfaction with aesthetic outcome was high. Notably, almost all subjects were satisfied with the natural appearance of the NLFs after correction, which is a common concern with aesthetic treatments.

Both devices were well tolerated. Most adverse effects were ISRs and were expected with injectable HA-based dermal fillers [5]. Although the incidence of adverse effects was slightly higher with STYL-L than JUV-3, this was not reflected in the subject satisfaction questionnaires. Mean ratings of pain at injection were low with both devices, confirming the benefit of the addition of anesthetic lidocaine to reduce pain.

A major strength of the NICE study was the double-blind, randomized design. The risk of bias was mitigated by blinding subjects and evaluators to the filler received. In addition, as there were several injectors from different countries, the results can be generalized. Furthermore, outcomes were both evaluator- and subject-reported. The use of subject-reported outcomes is particularly valuable in a study assessing aesthetic outcomes. Finally, another strength was the objective measuring of NLF depth using interference fringe projection profilometry. A limitation was the small subject number, particularly for the profilometry analysis, which was only available in one center. Injectors were not blinded; however, this limitation was mitigated as injectors did not assess effectiveness or safety.

## Conclusion

The NICE study demonstrated the non-inferiority of STYL-L versus JUV-3 in the improvement of NLFs at 6 months after treatment initiation. Results obtained up to 12 months suggest comparable effectiveness and safety profiles. Aesthetic improvement was reported in almost all subjects throughout the study and overall subject satisfaction was high with both devices.

## Data Availability

The data that support the findings of this study are available upon request.
